# Book Review: Éléments de Sociologie du Sport

**DOI:** 10.3389/fpsyg.2020.02004

**Published:** 2020-09-04

**Authors:** Bordes Pascal

**Affiliations:** ^1^UFR Sciences et Techniques des Activités Physiques et Sportives, Université Paris Descartes, Paris, France; ^2^URP3625 Institut des Sciences du Sport et de la Santé de Paris (I3SP), Paris, France

**Keywords:** sport, institution, traditional games, interaction network, score

The universe of physical games is exuberant; as it happens with languages, variety reigns, and diversity is the law. The great contribution of Pierre Parlebas's work is not only to remind us of this but more importantly to demonstrate it using a rigorous methodology linked to an innovative standpoint. According to the author, the study of sporting games must not only be related to classic ≪external≫ analyses: namely, sociological, psychological, anatomical, or physiological ones. It can also, and above all, emerge from a particular point of view that analyses from the inside what these practices are like and how they function. By explaining the game for its own sake, this approach, far from confirming the inherited ideas about sport, takes them off balance. In the wake authors such as Saussure, Bachelard, or Canguilhem, Parlebas builds up in the first place the research object of his project: sporting game. After dismissing and refusing literary dissertations, general assumptions, and obstructing truisms, he proposes a set of rigorous operational definitions that makes it possible to find order in the ≪wasted lands≫ he is confronted with.

Sport is therefore coherently conceived and understood as ≪the finite and countable set of motor situations codified in the form of institutionalized competition≫ (p. 55). It is from this definition that everything starts; it is from this point of view that the varieties of games emerge and their variations take shape. To start with the mode of resolution, sports situations are motor situations by nature; that is, situations that have no existence apart from that of the player's physical involvement. In other words, sports are situations in which ends and means merge, task and activity are one and the same. The motor action deployed by the players is limited to its very bodily realization, something that distinguishes it from other types of games whose pertinence is not of motor nature: card games, for example. The second trait allows us to differentiate unregulated, non-competitive physical practices, such as just jogging, and those which attest to rule-created confrontation, such as Athletics. Finally, the institutional trait completes the definition. Often overlooked, this trait becomes conclusive for finally distinguish sports from other ludomotor practices. Any traditional or street sporting game is precisely distinguished from sports because it lacks recognition by the authorities in place, federations and international committees in particular. On the contrary, as we know, sports embody massive hierarchical, centralized, and normative regulatory systems.

What can we learn after reconsidering what sport is from scratch? First of all, that among all the modalities that can be found in the universe of physical games, sport and its globalized practices only represent an over-published little island, a ≪restricted subset≫ of sporting games, as Parlebas puts it (p. 252). Our ludomotor heritage very much exceeds in number the sum of the recognized and accountable sports disciplines. Promptly, a second observation emerges. Behind the apparent diversity of forms in the sports world there operates one same deep structure: that which favors opposition in the form of duels of teams or individuals that fight on equal terms according to scores of capitalizable numerical units. This model is outrageously dominant, says Parlebas, for its simplicity facilitates quick reading and immediate understanding, feeding a spectacular dimension capable of mobilizing crowds regardless of their ethnic or socio-cultural backgrounds. Elementary in the way it functions, but very efficient when it comes to designate victors and vanquished, sport presents itself as the Esperanto of physical games.

Nonetheless, what sport gains in dissemination and accessibility is lost in richness and complexity: its universal claim is more that of *Globish*, the impoverished planetary English with a hegemonic vocation, a universal means of communication for the citizen of the world, with no past, no borders and no culture. Sport is that standardized ≪world game≫ with a uniformed, elementary functioning that puts in danger our cultural diversity, which is characteristic of the human species. Contrary to sport, traditional sporting games (that is, non-sportified games) display an insolent luxuriance, and Pierre Parlebas' merit is, first and foremost, bringing that to light. Concepts and analysis tools specially forged or redefined for the occasion make possible both to explore this diversity and put it in order, making it intelligible at the same time. For instance, the communication networks between players or teams are much more varied than what the sports' model exhibits: each one for themselves, compulsory rotation within the team or at the good will of the players, permutation of players or even asymmetrical confrontations in rights and duties… The relational palette of traditional sporting games goes far beyond the face-to-face situation of sports confrontation, and the same is true for the different roles that each agent may take on during game-playing. While sport reduces their number to a handful of positions from which performance must be pushed to the limit, traditional games multiply functions and attributions upon which players can move along. As far as scoring is concerned, sports only retain certain noteworthy acts, generally produced against the opponent, in the form of quantified capitalization. Pierre Parlebas shows that traditional games offer, here again, a multiplicity of scenarios: not all scores necessarily turn into accounting results, just as there are games with regressive accounting in which the reversal of roles resets the game to the starting situation. More surprisingly, many games operate without any scoring support, avoiding the designation of winners.

É*léments de sociologie du sport* is, in short, a fundamental work that clearly teaches that there is a difference in nature between sports and the so-called ≪traditional≫ games. In deep disdain for their intrinsic richness, sports imperialism tends to eliminate traditional games, often ≪more developed and “complicated” than that of institutional play≫ (249). Even though, as part of the ludocultural heritage of humanity these practices deserve not only attention and preservation but promotion and revaluation, because only they can offer alternative relational models to the reductionist competitive logic of the dominant sports model.

## Author Contributions

The author confirms being the sole contributor of this work and has approved it for publication.

## Conflict of Interest

The author declares that the research was conducted in the absence of any commercial or financial relationships that could be construed as a potential conflict of interest.

